# Does Erector Spinae Plane Block Have a Visceral Analgesic Effect?: A Randomized Controlled Trial

**DOI:** 10.1038/s41598-020-65172-0

**Published:** 2020-05-21

**Authors:** Hye-Mee Kwon, Doo-Hwan Kim, Sung-Moon Jeong, Kyu Taek Choi, Sooin Park, Hyun-Jung Kwon, Jong-Hyuk Lee

**Affiliations:** Department of Anesthesiology and Pain Medicine, Asan Medical Center, University of Ulsan, College of Medicine, Seoul, 05505 Korea

**Keywords:** Health care, Medical research

## Abstract

The visceral analgesic efficacy of erector spinae plane block (ESPB) is still a matter of debate. This study attempted to investigate the visceral analgesic efficacy of ESPB in clinical setting. After randomized, we performed ultrasound-guided bilateral rectus sheath block (RSB), which was aimed to prevent postoperative somatic pain on all patients who underwent laparoscopic cholecystectomy (LC). Ultrasound-guided bilateral ESPB at T7 level was performed only to the intervention group to provide the visceral analgesic block. The intraoperative requirement for remifentanil (P = 0.021) and the cumulative fentanyl consumption at postoperative 24-hours was significantly lower in the ESPB group (206.5 ± 82.8 μg vs.283.7 ± 102.4 μg, respectively; P = 0.004) compared to non-ESPB group. The ESPB group consistently showed lower accumulated analgesic consumption compared with those in the non-ESPB group at all observed time-points (all P < 0.05) after 2 hours and the degree of the accumulated analgesic consumption reduction was greater (P = 0.04) during the 24-hour postoperative period. Pain severity was lower in the ESPB group at 6-hours postoperatively. The significantly reduced opioid consumption in ESPB group may imply that while preliminary and in need of confirmation, ESPB has potential visceral analgesic effect. Therefore, performing ESPB solely may be feasible in inducing both somatic and visceral analgesia.

## Introduction

Since its first description in 2016 by Forero *et al*.^1^, erector spinae plane block (ESPB) has been the focus of attention as an alternative analgesic method. Targeting the space between the erector spinae muscle sheath and the transverse process of a vertebra, the injected agent spreads craniocaudally, resulting in the blockage of multiple vertebral levels, covering a wide area. Of note, it penetrates anteriorly into the paravertebral space where it can theoretically block not only the dorsal and ventral rami, but also the rami communicantes^2,3^, which suggests the potential of both somatic and visceral pain blockage.

Laparoscopic cholecystectomy (LC), although minimally invasive compared to open surgery, is associated with significant levels of postoperative pain^4^. The components of acute postoperative pain are consisted of somatic, visceral, and referred pain. Visceral pain is considered to be the co-dominating pain component, along with somatic pain, within 24 hours after LC^5,6^. Furthermore, early visceral pain is associated with an unexplained chronic pain development 1-year after LC^7^.

In this setting, performing ESPB as postoperative analgesic method may efficiently provide somatic and visceral pain block and overcome the current limitations of neuraxial and peripheral regional block. It is considered as a peri-paravertebral regional analgesia technique with a mechanism of action similar to that of the paravertebral block; however, it is technically easier and has lower risk of major complications, such as pneumothorax or accidental neuraxial injection^8^. For these reasons, ESPB is gaining popularity quickly despite it being a recently introduced technique. To date, its analgesic efficacy has been described in abundance in case reports^9^; however, only few controlled clinical trials after abdominal^10,11^, cardiac^12,13^, and breast surgery^14^ have been reported. Few cadaveric studies have showed that the injected dye penetrates to paravertebral space and spread into rami communicantes^2,3^, however, controlled clinical studies evaluating the visceral analgesic efficacy of ESPB have not been performed. In the previous study by our group, the preoperative rectus sheath block (RSB) effectively reduced the analgesic requirement^15^, however most of the patients complained the residual visceral pain, referring it as “pain from inside”.

Therefore, we designed this randomized, single-blind trial to investigate the efficacy of ESPB in visceral pain analgesia. Specifically, we performed RSBto prevent somatic sensory pain in patients undergoing LC and hypothesized that ESPB may provide additional pain relief by alleviating the visceral pain. The primary outcome was to assess the effect of ESPB on the cumulative consumption of a rescue analgesic over 24 postoperative hours. The secondary outcomes were the intraoperative remifentanil requirement and postoperative pain score.

## Results

**Figure 1:**
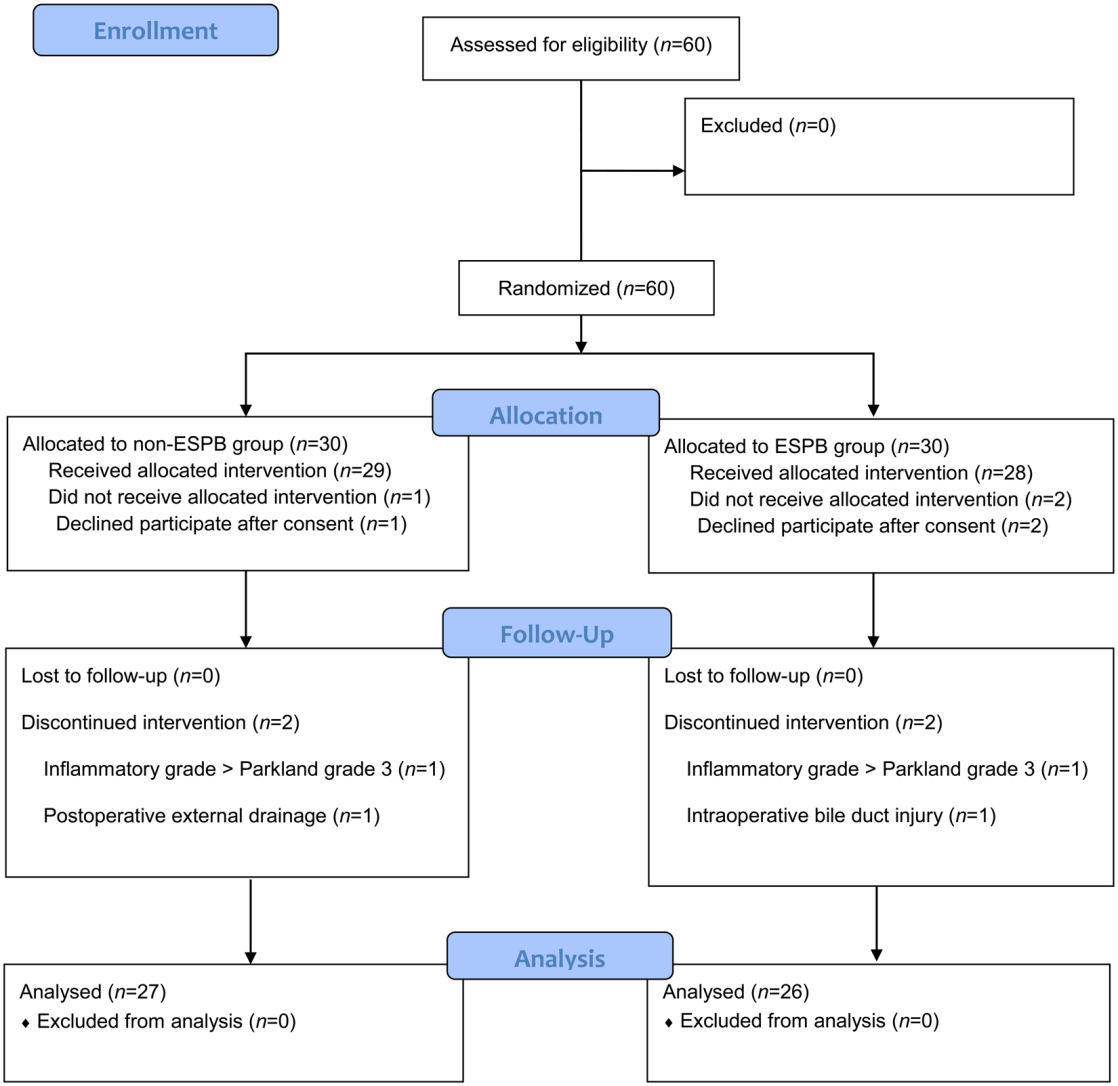
Study flow diagram of the participants. RSB, rectus sheath block, ESPB, erector spinae plane block.

A total of 60 patients were enrolled and 53 were included in the final analysis (Fig. 1). Of all, 7 patients were excluded. Two patients were excluded due to high severity grade of the cholecystitis (>Parkland grade 3), one patient due to intraoperative bile duct injury, one patient due to postoperative external drainage, and three patients due to refusal. Finally, 27 patients in non-ESPB group and 26 patients in ESPB group were analysed. There were no significant differences in the demographics between the groups, including the pre-operative diagnosis (Table 1). The cholecystitis inflammation status evaluated by the surgeon through visual inspection (P = 0.111), rate of intraoperative bile leakage incidences (P = 0.973), grade of gallbladder injury (P = 1.000), peak abdominal gas pressure (P = 0.188), and duration of the surgery (P = 0.551) were not different between the groups (Table 2 and Supplementary Table 1). In addition, there was no significant difference between the changes from baseline to maximum mean blood pressure (P = 0.144) and heart rate (P = 0.110) collected within 5 minutes after skin incision and prior to pneumoperitoneum. None of the patients reported predominating residual somatic resting pain at the PACU.

**Table 1:** Patients demographics.

	Non-ESPB group (n = 27)	ESPB group (n = 26)	P value
Age (y)	48.2 ± 11.8	51.5 ± 11.7	0.310
Sex (male, %)	12 (44.4%)	9 (34.6%)	0.665
Height (cm)	163 (157–175)	162 (156–171)	0.544
Weight (kg)	67.1 ± 14.2	66.9 ± 10.0	0.945
Body mass index (kg/m^2^)	24.5 ± 3.2	25.0 ± 3.1	0.588
ASA (1/2)	12/15	12/14	1.000
Diabetes mellitus	3 (11.1%)	2 (7.7%)	1.000
Hypertension	10 (37.0%)	8 (30.8%)	0.848
Diagnosis			0.694
*Cholecystitis	21 (77.8%)	18 (69.2%)	
+Others	6 (22.2%)	6 (30.8%)	
Preoperative pain score, (0/1, NRS)	25/2	26/0	0.488

Values are expressed as the mean (±SD) or median (interquartile range) for continuous variables, and n (%) for categorical variables. *Cholecystitis: Preoperatively diagnosed as acute or chronic cholecystitis; +Others: Preoperatively diagnoses other than cholecystitis, which includes gallbladder stone, gallbladder polyp, and adenomyomatosis. NRS: numeric rating scale.

**Table 2:** Intraoperative data.

	Non-ESPB group (n = 27)	ESPB group (n = 26)	P value
**Intraoperative findings**			
Inflammation grade (1/2/3)^*^	9/16/2	14/8/4	0.111
Gallbladder bed injury (1/2/3)^**^	25/2/0	25/1/0	1.000
Duration of operation (min)	27.0 ± 10.2	28.8 ± 12.6	0.551
Intraoperative remifentanil use (μg/min/kg)	0.073 ± 0.017	0.063 ± 0.015	0.024
**Difference between maximum value and baseline vital signs**			
Mean blood pressure (mmHg)	−1.1 ± 3.9	0.2 ± 2.7	0.144
Heart rate (beats/min)	−1.0 (−1.0–0.5)	−1.0 (−3.0–0.0)	0.110

Data presented as n (%) the mean(±SD) or median (interquartile range) for continuous variables, or absolute number for categorical variables, as appropriate.

*Evaluated by Parkland grading method.

**Evaluated by operating surgeon as follows: 1. Insignificant injury to liver, 2. Mild injury to liver 3. Moderate injury to liver.

**Figure 2:**
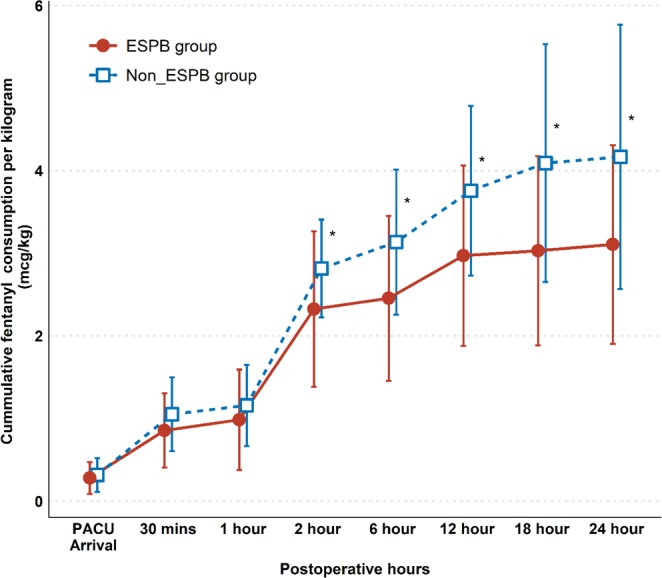
Cumulated analgesic consumption. All analgesics were converted to equivalent fentanyl dose (μg) and were divided by patient’s weight (kg). Cumulated fentanyl consumption at 24-hour is the primary outcome. Data are expressed as the median and interquartile range. ∗P < 0.05 indicates statistical significance. ESPB, erector spinae plane block; PACU, postanaesthetic care unit.

The intraoperative requirement for remifentanil was significantly lower in the ESPB group compared to that in the non-ESPB group (P = 0.024, Table 2). After discharge from the PACU, patients in the ESPB group had lower accumulated analgesic consumption compared with those in the non-ESPB group (Fig. 2).

Of note, repeated measures analysis revealed that the degree of accumulated analgesic consumption reduction was greater (P = 0.04) during the 24-hour postoperative period. The mean difference in cumulative analgesic consumption at 6 postoperative hours was 41.9 mcg (rate of difference, 25.3%; 165.1 ± 67.7 mcg vs 207 ± 45.5 mcg, P = 0.012), which significantly increased to 77.2 mcg (rate of difference, 37.4%; 206.5 ± 82.8 mcg vs. 283.7 ± 102.4 mcg, P = 0.004) at 24 postoperative hours.

The NRS score at 6 hours postoperatively was significantly lower in the ESPB group than in the non-ESPB group (2 (2-3) vs. 3 (3-5), P = 0.029); however, there was no difference in the NRS scores between the other time points (Table 3). The incidences of postoperative nausea and vomiting were not different between the two groups (Table 4). None of the patients reported any signs of local anaesthetic toxicity or other adverse effects associated with local anaesthetic administration during or after surgery.

**Table 3:** NRS after surgery.

Time after surgery	Non-ESPB group (n = 27)	ESPB group (n = 26)	P value
0 hour	5 (4–6)	5 (2–6)	0.737
0.5 hour	6 ± 2	5 ± 2	0.225
1 hour	3 (2–3)	3 (2–3)	0.277
2 hours	5 (4–7)	5 (4–6)	0.453
6 hours	3 (3–5)	2 (2–3)	0.019
12 hours	2 (1–4)	3 (2–4)	0.196
18 hours	2 (2–3)	2 (2–3)	0.872
24 hours	2 (1–3)	2 (1–3)	0.792

Values are expressed as the mean (±SD) or median (interquartile range). NRS, Numeric rating score. Postoperative 0, 0.5, and 1 hour are measured in the recovery room.

**Table 4:** Postoperative Complications.

Side effects or Complications	Non-ESPB group (n = 27)	ESPB group (n = 26)	P value
**Incidence of Nausea**			
PACU	1 (4%)	2 (8%)	0.973
General ward (within 24 hours)	4 (14.8%)	2 (7.7%)	0.701
**Incidence of Vomiting**			
PACU	0 (0.0%)	1 (4%)	0.985
General ward (within 24 hours)	1 (3.7)	1 (3.8)	1.000
**Rescue mediation for nausea or vomiting**			
PACU	0 (0.0%)	2 (8%)	0.454
General ward (within 24 hours)	5 (18.5)	1 (3.8)	0.211

Values are expressed as n (%). PACU, post-anaesthetic care unit.

## Discussion

In this randomized, single-blind clinical trial, we demonstrated that analgesia with ESPB and RSB reduced the cumulative analgesic consumption for up to 24 hours, with the degree of reduction being more pronounced compared to analgesia with RSB alone. The intraoperative opioid requirement was also lower in the ESPB group than in the non-ESPB group. The 14% reduction during surgery and 37% reduction after surgery in opioid usage were found in the ESPB group, compared to non-ESPB group. The incidences of postoperative nausea and vomiting were similar in both groups. Our results suggest that ESPB may be effective in visceral sensory blockage.

ESPB has emerged as a possible solution to the current limitations to the existing analgesic methods. The target site for injection is the space between the erector spinae muscle sheath and the transverse process of the vertebra, which reduces the risk of needle-pleura interaction and possible pneumothorax. As the injected agent seems to spread not only craniocaudally, but also anteriorly into paravertebral area, ESPB is thought to provide analgesia both for several dermatomes at the level of injection and is considered to also have a potential to block somatic and visceral pain. Since the first description in 2016 by Forero *et al*.^1^, its clinical indication has broadened by adjusting the injection target between T5 to L1 according to the targeting site. As a result, the popularity of ESPB grew quickly despite of it being introduced recently. Currently, numerous case reports have continuously demonstrated its analgesic effect and recently, a few controlled trials were reported. Currently it is evident that the ESPB is effective with its major advantages, such as potential for visceral pain blockage, simpler technique, and fewer risks of complication. Thus, ESPB could be the alternative method to classic neuraxial and paravertebral block. However, although theoretically possible, effectiveness of ESPB in visceral sensory blockage is still controversial.

Although LC is minimally invasive compared with open surgery, this procedure is still associated with significant levels of postoperative pain^4^, which prolongs the hospital stay and increases readmission rates^16^. The postoperative pain is the worst in the first 24 hours after surgery, with visceral pain being the predominant pain, followed by somatic pain^5,6^. Furthermore, patients with higher visceral pain intensity showed higher risk of a chronic pain development after LC.

In this regard, effective postoperative pain management has been actively researched^17,18^ for it is essential in patient care. Currently, a multimodal approach is recommended to reduce the opioid consumption and associated side-effects^17,19^. Neuraxial regional analgesia, such as paravertebral block^20^, thoracic epidural block^21^, or spinal anaesthesia^22^, are effective in both visceral and somatic pain blockage. However the current consensus does not support neuraxial analgesia for use in patients undergoing minimally invasive surgery such as LC^18^, due to the potential complications related to the procedure, such as pneumothorax, direct spinal cord injury, epidural hematoma, and central infection. The benefit of neuraxial analgesia does not seem to outweigh the potential risks, and importantly, they seem unnecessary in the context of minimally invasive surgery in patients using laparoscopic procedures^8,18,23^. Peripheral regional blocks, such as rectus sheath block^15,24^ or transversus abdominis block^25^, could be alternative analgesic methods^18^ with the advantage of being relatively safe procedures with high efficacy^26^. However, these have limitations for being effective only in blocking somatic pain.

In previous studies regarding patients undergoing LC, the ESPB successfully provided analgesia compared to the placebo group^11^. ESPB reduced intra- and post-operative opioid consumption and lowered the pain score^27^. Moreover, it was superior to the oblique subcostal transversus abdominis plane block^28^ in postoperative pain control. Our study is the first controlled clinical study to evaluated the efficacy of the ESPB in terms of visceral pain blockage.  Our result shows that the ESPB further reduces analgesic consumption in patients with reduced somatic pain via RSB, compared to those with only RSB. Of note, the amount of  the reduced analgesic consumption was greater over the observed time period. It can be assumed that the ESPB has provided pre-emptive analgesic effects for the visceral pain, which led to peripheral desensitization resulting in reduced opioid consumption.

The mechanism of action in ESPB has been studied in a few cadaveric studies with conflicting results. In 2016, the study by Forero *et al*. was first to investigate the spread of local analgesics with two cadavers, and showed the anterior spread through the paravertebral space, resulting in blockage of not only ventral and dorsal rami but also rami communicantes, showing the potential of visceral sensory blockage. In another study by Yang *et al*., the injected dye penetrated anteriorly into the paravertebral space, similarly as in the previous study^2,3^. However, in the study by Ivanusic *et al*., the injected dye spread only craniocaudally and laterally to the posterior of the costotransverse foramen, and no anterior spread into the dorsal and ventral rami was found^29^. In clinical settings, one case report described the potential visceral analgesic effect of ESPB in bariatric surgery^30^. A very recent randomized controlled study reported that ESPB provided effective analgesia in patients undergoing open epigastric hernia repair with midline incision^31^. Taken together, these clinical studies suggest the anterior spread of the local agent into the paravertebral space in ESPB. It could be speculated that the area of spreading may be different in cadaveric patients than in the living ones due to the intrathoracic pressure change and the absence of tissue tension in living patients.

Our study has several limitations. First, we did not check the sensory distribution of ESPB. One of the reasons is the postoperative hyperaesthetic state of the abdomen due to the pneumoperitoneum, and the other is that the use of opioid after surgery may have changed the pain threshold. Taken together, the exact dermatome by pin-prick test would not be feasible. However, we visually confirmed that the local anaesthetics were well spread in the transverse and the sagittal view in all patients receiving ESPB with ultrasound, therefore we may speculate that ESPB was well performed. Second, we did not provide somatic pain block for the epigastric and subcostal 5 mm trocar area. However, it is reported that the port size is related to pain and 5 mm is shown to induce less postoperative pain after laparoscopic surgery^32^. Furthermore, none of our patients reported predominating resting somatic pain on zyphoid or subcostal area at the PACU. Third, although the fentanyl consumption is statistically significant, the absolute dosage may not clinically significant (3 mcg/hr) and there is no difference in NRS between the two groups. In this study, the RSB was performed in all patients to prevent the somatic pain, which reduced postoperative pain and decreased opioid consumption in the control group^15^. In addition, because of rescue analgesics was administered when NRS was ≥ 4 or when the patient needed pain relief, we thought that the NRS was not significantly difference between two groups. “Due to the design of the current study, routine analgesia was not provided but only upon the patients’ request or NRS ≥ 4, which may have resulted relatively higher NRS score in current study compared to the data reported by previous studies. Although ESPB is an effective analgesia approach especially in the first 6-8 hours, the patients should receive routine analgesia not to develop a pain-memory”. Furthermore, based on these reasons, the current study does not justify using two different blocks in patients undergoing LC. The value of our results lies that it is the first to report the effectiveness of ESPB focusing on the visceral analgesic effect in clinical setting, which may assist broadening the ESPB indication in perioperative pain management.

## Conclusions

Ultrasound-guided ESPB with RSB reduced the intraoperative remifentanil requirement and cumulative analgesic consumption in the first 24 hours in patients undergoing LC compared to that in patients using RSB only, suggesting the potential visceral analgesic effect of ESPB. Although further clinical studies are needed to clarify the extent of analgesia, our results show that ESPB may be an effective technique for the management of postoperative visceral pain.

## Materials and Methods

This is a single-center, prospective, randomised, single-blind trial. All subjects gave their informed consent for inclusion before they participated in the study. The study was conducted in accordance with the Declaration of Helsinki, and the protocol was approved by the Ethics Committee of the Institutional Review Board of Asan Medical Center, Seoul, Republic of Korea (2018-1320). This study was registered on December 07, 2018 at clinicalTrials.gov (NCT03767816) prior to patient enrolment.

### Patients

Patients scheduled for elective LC between December 17, 2018 and January 29, 2019 were screened for eligibility. Patients aged 20–80 years with an American Society of Anesthesiologists physical status class 1 or 2 were eligible for the study. Exclusion criteria were as follows: contraindications for regional anaesthesia, such as history of local anaesthetic allergy or steroid complication; use of anticoagulants; pregnancy or breastfeeding; history of previous abdominal surgery; pre-existing vertebra or chest wall abnormality; and refusal to participate. The study also excluded patients with severe intraperitoneal inflammation or adhesions of cholecystitis (Parkland grade > 3)^33^, those with a single port insertion, patients with intraoperative bile duct injury, and those who maintained percutaneous drainage after surgery. Patients were randomized into two groups (ESPB group and non-ESPB group) according to a computer-generated randomization schedule created before the study start. Patients in the ESPB group received bilateral ESPB before general anaesthesia induction, whereas bilateral RSB were performed immediately after anaesthesia induction. In non-ESPB group, only bilateral RSB were performed immediately after induction and prior to incision. The regional blocks and anesthesia were performed by one of two investigators (H.-M. Kwon or J.-H. Lee). Subsequent data collection was performed by the blinded study research coordinator or a blinded study investigator.

### Ultrasound-guided ESPB and RSB

ESPB was performed according to a standardized method, as previously described by Forero *et al*.^1^. After placing the patient in a prone position, a NextGen LOGIQe ultrasound console (GE Healthcare, Madison, WI, USA) with a 12 MHz high-frequency linear ultrasound transducer was placed in a longitudinal orientation 3 cm lateral to the T7 spinous process. Since ribs articulate posteriorly with the corresponding thoracic vertebra, we visualized the 12^th^ rib and counted from there to the 7^th^ rib to confirm the location of T7 thoracic vertebra. After identifying the fascia of erector spinae muscle superficial to the tip of the transverse process, under aseptic conditions, a 22-gauge Quincke needle (TaeChang Industrial Co., Gongju, Korea) was inserted in a caudal-to-cephalad direction by in-plane technique, until the needle tip touched the tip of the transverse process of T7 vertebrae and was laid in the fascial plane on the deep aspect of the erector spinae muscle. The location of the needle tip was confirmed by a visible linear spread of fluid lifting the erector spinae muscle off the transverse process on ultrasonographic imaging. A total of 20 mL of 0.20% ropivacaine was injected on one side. The same procedure was performed with 20 mL of 0.20% ropivacaine on the contralateral side.

RSB was performed after identifying the rectus abdominis muscle by placing the ultrasound probe in a transverse orientation next to the incision site, which was 1 cm below the umbilicus, and a 22-gauge Quincke needle (TaeChang Industrial Co., Gongju, Korea) was inserted in-plane, medial-to-lateral direction to place the needle tip in the plane between the lateral side of the rectus abdominis muscle and the posterior rectus sheath. After confirming the needle tip by visible linear spread lifting the rectus sheath muscle, 15 mL of 0.20% ropivacaine was administered, and the same procedure was repeated on the opposite side.

### Surgical technique of LC

LC was performed using a three-port surgical technique according to our hospital protocol. In all patients, a 12 mm port was placed at the 1 cm below umbilicus, 5 mm port in the epigastric area, and another 5 mm port was placed on the right subcostal area. Non-humidified and non-heated CO2 were used to achieve pneumoperitoneum and the intra-abdominal pressure was maintained to be lower than 13 mm Hg in all patients.

### Anaesthesia and analgesia

In the operating room, routine monitoring included electrocardiography, non-invasive blood pressure measurement, and pulse oximetry. After induction of anaesthesia using propofol (2 mg/kg), rocuronium (0.6 mg/kg) was administered and endotracheal intubation was performed. Balanced anaesthesia was maintained using desflurane (5–6%) in 50% oxygen and a continuous infusion of remifentanil (1.5–2.5 ng/ml of effect-site concentration, using Orchestra, Fresenius Vial, France). Remifentanil was maintained under 2.7 ng/ml to avoid postoperative hyperalgesia^34^. Changes of the intraoperative blood pressure were maintained within 20% from the baseline values. After emergence from anaesthesia, patients were transferred to the post-anaesthetic care unit (PACU) where pain was assessed and recorded by nursing staff using an 11-point Numerical Rating Scale (NRS). Intravenous fentanyl bolus (0.4 µg/kg) was administered upon patients’ request or when analgesia was insufficient (NRS ≥ 4). Administration of fentanyl was repeated until NRS < 4 or the patient did not request further pain relief. After transferring to the general ward, 50 mg of dexketoprofen, and 50 mg of tramadol or 25 mg of meperidine were administered in sequence only upon patient request or NRS was ≥ 4.

### Outcome measures and data collection

The primary outcome was the difference in the 24-hour postoperative cumulative rescue analgesic consumption between patients in the ESPB and non-ESPB group. Secondary outcomes were intraoperative remifentanil consumption and postoperative pain score, evaluated by NRS.

The baseline pain level prior to surgery was evaluated by a single investigator to exclude any underlying visceral pain. Because this study was intended to evaluate the efficacy of ESPB in alleviating visceral pain, the patients with predominant somatic pain after the RSB were exclude from further analysis. Given that objectively differentiating pain component is difficult in clinical setting, an investigator, who was blinded to the treatment allocation, asked the patients if they had any ‘resting superficial pain’ and further confirmed by giving pressure on the umbilical port incision site to check for any residual resting somatic pain at PACU. Postoperative pain intensity was assessed using a single 11-point NRS (in which 0 = no pain and 10 = worst pain imaginable). Nursing staff blinded to the randomization administered intravenous rescue analgesics and recorded the total doses of rescue analgesics during the 24-hour postoperative period. The doses of all opioids and nonsteroidal anti-inflammatory drugs administered to patients were converted to intravenous fentanyl equianalgesic doses based on previously published conversion factors (intravenous fentanyl 100 μg = ketorolac 30 mg = tramadol 100 mg)^35^. Cumulative analgesic consumption and the NRS were measured at 0, 0.5-, and 1 h at the PACU, and at 2-, 6-, 12-, 18- and 24-h at the general ward after the surgery.

Additional factors associated with visceral pain, such as the severity of adhesions and the cholecystitis inflammation status were graded according to the Parkland scale (range 0–5)^33^ by the operating surgeon based on visual inspection. The surgical procedure characteristics related to visceral pain, such as the duration of surgery, peak abdominal gas pressure, severity of gallbladder bed injury during surgery, or rate of intraoperative bile leakage, were also compared between the groups. The severity of gallbladder bed injury was reported by the surgeon as follows: 1. insignificant injury to the liver; 2. mild injury to the liver; and 3. moderate injury to the liver. Vital signs were recorded, such as mean blood pressure (mm Hg) and heart rate (beats per minutes), measured before the incision, and the maximum value after the incision and before the induction of pneumoperitoneum. Furthermore, the adverse effects of analgesics, such as nausea and vomiting, and the complications associated with ESPB or RSB procedure, such as hematoma or pneumothorax, if any, were also recorded.

### Sample size calculation

Based on our previous study^15^, we expected that the postoperative 24-hour cumulative fentanyl consumption would be 220 ± 86 μg in patients receiving RSB. Sample size estimation, with a power of 80% and an alpha error of 0.05, showed that 25 patients in each group would be needed to reach significance of the 20% reduction of the 24-hour fentanyl consumption in patients receiving RSB and ESPB. To be able to compensate for an incomplete data collection or patients dropping out, a total of 60 patients were recruited.

### Statistical analysis

Continuous parameters were summarized as mean (± standard deviation) or median (interquartile range), and categorical parameters as frequency (percentage), as appropriate. Between-group comparisons were evaluated using the Student’s t-test or Mann–Whitney U-test for continuous variables, and the Chi-square or Fisher’s exact test for categorical variables, as appropriate. The repeated measurements of cumulative fentanyl consumptions were performed using a linear mixed-effect model to evaluate the interaction of time and treatment between the ESPB and non-ESPB groups. Statistical significance was set at P < 0.05. Data manipulation and analyses were performed using R software, version 3.6.2 (CRAN, R Foundation, Vienna, Austria)^36^

## Supplementary information

Supplementary Information.
