# Supplementary material for: Does Erector Spinae Plane Block Have a Visceral Analgesic Effect?: A Randomized Controlled Trial
Source: Sci Rep. 2020 May 21;10:8389. doi: 10.1038/s41598-020-65172-0 (PMC7249264; doi:10.1038/s41598-020-65172-0)
Supplement: Supplementary file 1 — Supplementary Information. [file 41598_2020_65172_MOESM1_ESM.docx]

*Does* Erector Spinae Plane Block Have a Visceral Analgesic Effect?: A Randomized Controlled Trial

Hye-Mee Kwon, PhD, Doo-Hwan Kim, MD, Sung-Moon Jeong, PhD, Kyu Taek Choi, PhD, Sooin Park, MD, Hyun-Jung Kwon, MD, and Jong-Hyuk Lee*, PhD

**From the**

Department of Anesthesiology and Pain Medicine, Asan Medical Center, University of Ulsan, College of Medicine, Seoul 05505, Korea

**Supplementary Table 1.** Intraoperative data regarding the surgical procedure

|  | **Non-ESPB group**  **(n = 27)** | **ESPB group**  **(n = 26)** | **P value** |
| --- | --- | --- | --- |
| Intraoperative findings | |  |  |
| Intraoperative bile leakage | 1 (3.7%) | 2 (7.7%) | 0.973 |
| Peak abdominal gas pressure (11/12/13) | 1/24/2 | 0/20/6 | 0.188 |

Data are expressed as n (%) or the absolute number, as appropriate.
